# Supplementary material for: Village-scale persistence and elimination of gambiense human African trypanosomiasis
Source: PLoS Negl Trop Dis. 2019 Oct 28;13(10):e0007838. doi: 10.1371/journal.pntd.0007838 (PMC6837580; doi:10.1371/journal.pntd.0007838)
Supplement: S1 Appendix — Additional information on the formulation of the model, details of the data and supplementary results. (PDF) [file pntd.0007838.s001.pdf]

# Supporting Information
# Village-scale persistence and elimination of *gambiense* human African trypanosomiasis

Christopher N. Davis[1,2], Kat S. Rock[2,3], Erick Mwamba Miaka[4], Matt J. Keeling[2,3,5*]

* Corresponding author: M.J.Keeling@warwick.ac.uk
1 MathSys CDT, Mathematics Institute, University of Warwick, Coventry, CV4 7AL, UK
2 Zeeman Institute (SBIDER), University of Warwick, Coventry, CV4 7AL, UK
3 Mathematics Institute, University of Warwick, Coventry, CV4 7AL, UK
4 Programme National de Lutte contre la Trypanosomiase Humaine Africaine (PNLTHA), Ave Coisement Liberation et Bd Triomphal No 1, Commune de Kasavubu, Kinshasa, DRC
5 School of Life Sciences, University of Warwick, Coventry, CV4 7AL, UK

## Model

### Model equations

We introduce a compartmental infection model for HAT. The outputs of the model are the number of humans and the proportion of the total number of vectors in each class. For humans, the classes are: susceptible, exposed (but not infectious), Stage 1 infection, Stage 2 infection and hospitalised (or recovering at home). These variables are denoted by $S_{Hj}(t)$, $E_{Hj}(t)$, $I_{1Hj}(t)$, $I_{2Hj}(t)$ and $R_{Hj}(t)$, where $j = 1, 2$, with $j = 1$ for low risk individuals, randomly participating in active screening, and $j = 2$ for high risk individuals, never participating in active screening, each at time $t > 0$. The population size is assumed to be constant (see Fig 7 for different assumptions on the populations) and thus

$$N_{Hj} = S_{Hj}(t) + E_{Hj}(t) + I_{1Hj}(t) + I_{2Hj}(t) + R_{Hj}(t), \qquad j = 1, 2.$$

The vectors (tsetse) are given by the variables $S_V$, $E_{1V}$, $E_{2V}$, $E_{3V}$, $I_V$ and $G_V$, which correspond to teneral (unfed) tsetse, infected tsetse in their extrinsic incubation period (there are three classes to create a gamma distributed period), infectious tsetse, and non-teneral (fed) but uninfected tsetse. The compartments of the model and possible transitions between them are shown graphically in Fig 1. We assume that there is natural mortality from all compartments, which leads to replacement of that individual as a susceptible in the population.

Active screening has the potential to detect infection and so move individuals from the exposed or infected classes directly to hospitalised. This is simulated by randomly selecting from the low risk population and if these individuals are exposed or infected, they become hospitalised with 91% probability (the sensitivity of the screening algorithm), as this is the probability of a positive test result, given the person has the infection.

For $m = m_{\text{eff}}/p_H$ and $p_H > 0$, Table 1 defines the transition rates of the Markov-chain model $\{(S_{H1}(t), E_{H1}(t), I_{1H1}(t), I_{2H1}(t), R_{H1}(t), S_{H2}(t), E_{H2}(t), I_{1H2}(t), I_{2H2}(t), R_{H2}(t), S_V(t), E_{1V}(t), E_{2V}(t), E_{3V}(t), I_V(t), G_V(t)) : t > 0\}$ from state $(s_{H1}, e_{H1}, i_{1H1}, i_{2H1}, r_{H1}, s_{H2}, e_{H2}, i_{1H2}, i_{2H2}, r_{H2}, s_V, e_{1V}, e_{2V}, e_{3V}, i_V, g_V)$. For $m \to \infty$ and $p_H \to 0$ (when the vector-to-host ratio, V:H $\to \infty$),

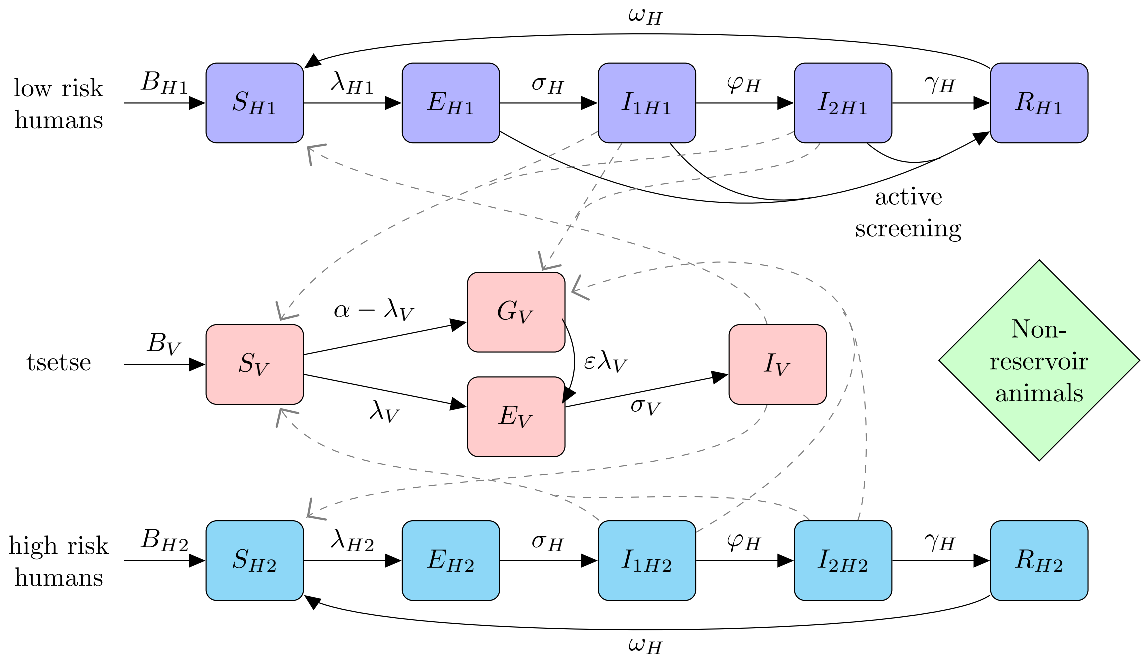


**Fig 1. Compartmental model of HAT infection dynamics in humans (low and high risk) and tsetse.** Humans can become exposed, $E_H$, on a bite from an infective tsetse and progress through the infection stages, $I_{H1}$ and $I_{H2}$, before moving to the non-infectious class $R_H$ due to hospitalisation. The birth rates $B_{Hi}$ are given by $\mu_H N_{Hi}$ for $i = 1, 2$ and $B_V$ is equal to $\mu_V N_H$. Active screening moves exposed or infected people directly to the hospitalised class. Unfed tsetse can become exposed and infected, $E_V$ and $I_V$, on consumption of a blood-meal, of which a proportion are taken on humans. Alternatively, first blood-meals not resulting in exposure means tsetse are less susceptible to trypanosomes in future meals, $G_V$, where we define $\lambda_V = \alpha p_V \left( f_{H1} \frac{I_{1H1}+I_{2H1}}{N_{H1}} + f_{H2} \frac{I_{1H2}+I_{2H2}}{N_{H2}} \right)$. The transmission of infection between humans and tsetse is shown by grey paths. Infected animals are not considered.

the transition rates remain the same in the human population, but now, since we are considering an infinite number of vectors, an ordinary differential equation approximation is used to describe the vectors. In simulating the model, the tau-leaping algorithm is used, whereby the states and rates are updated with a fixed time step at which the ordinary differential equations are also updated.

In our analysis, the endemic disease equilibrium is calculated as the steady state of the deterministic model, excluding control measures other than basic passive surveillance [8]. Initial conditions for individual villages are taken by sampling from a binomial distribution, where the probability of being selected in a class is given by the proportion in that class in the steady state of the model. Using random initial conditions with the mean at endemic equilibrium removes the effects of rounding the initial conditions to the same integer values for each simulation.

A sample realisation of the infection dynamics in a population with three active screening events, where randomly sampled infected low risk individuals are moved directly into the hospitalised class, is shown in Figure 2.

## Parameters

Table 2 defines the parameters used in the model for HAT infection dynamics. These parameter values are taken from Rock et al. [8], which were either sourced from literature, where well-defined, or otherwise (in the case of $m_{\text{eff}}, R_1, R_2, r$ and $u$) taken as the median of the distribution obtained by model fitting using a Metropolis–Hastings MCMC algorithm that matched the deterministic version of the model to incidence data from the WHO HAT Atlas.

Since the human population is partitioned into low and high risk individuals, we also denote $N_{H1} = N_H R_1$ and $N_{H2} = N_H R_2$, where $R_1 + R_2 = 1$. Similarly, $f_{H1} = f_H R_1/(R_1 + rR_2)$ and $f_{H2} = f_H r R_2/(R_1 + rR_2)$ to give the proportion of blood meals on each of the two risk groups, which sum to the total proportion of blood meals tsetse take on humans, $f_H$. Furthermore, we note that the effective density of tsetse is given by the product of the relative tsetse density and the probability of human infection per single infective bite, $m_{\text{eff}} = mp_H$.

## Risk structure

The risk structure in the human population is included, since there is evidence for differences in screening attendance and tsetse exposure within the population [5]. Furthermore, using deviance information criterion (DIC) as the method of model selection, there was strong evidence for the inclusion of risk structure [8]. Figure 3 shows the comparison of the models with the reported incidence data. The marginal improvement of the fit when an animal reservoir was added to the model meant animals were not considered here. The DIC was calculated by the following:

$$\text{DIC} = -2\text{LL}\left(\bar{\theta}\right) + 4\text{Var}\left(\text{LL}\left(\theta\right)\right),$$

where LL is the log-likelihood of unknown parameters $\theta$, and where $\bar{\theta}$ is the expectation of these parameters.

**Table 1. Model formulation.** The transition rates of the Markov-chain gHAT-infection model and additional ordinary differential equation component of the model when $m \to \infty$ and $p_H \to 0$ (V:H $\to \infty$).

| Class | Event | Transition | Rate |
|---|---|---|---|
| Humans, $j = 1, 2$ | Recovery from hospitalisation | $s_{Hj} \to s_{Hj} + 1, r_{Hj} \to r_{Hj} - 1$ | $\omega_H r_{Hj}$ |
| | Natural death of hospitalised | $s_{Hj} \to s_{Hj} + 1, r_{Hj} \to r_{Hj} - 1$ | $r_{Hj}$ |
| | Exposure of susceptibles | $s_{Hj} \to s_{Hj} - 1, e_{Hj} \to e_{Hj} + 1$ | $f_{Hj} \alpha p_H s_{Hj} i_V / N_{Hj}$, $p_H > 0$<br>$f_{Hj} \alpha m_{\text{eff}} s_{Hj} I_V / N_{Hj}$, $p_H \to 0$ |
| | Progression to Stage 1 infection | $e_{Hj} \to e_{Hj} - 1, i_{1Hj} \to i_{1Hj} + 1$ | $\sigma_H e_{Hj}$ |
| | Natural death of exposed | $s_{Hj} \to s_{Hj} + 1, e_{Hj} \to e_{Hj} - 1$ | $\mu_H e_{Hj}$ |
| | Progression to Stage 2 infection | $i_{1Hj} \to i_{1Hj} - 1, i_{2Hj} \to i_{2Hj} + 1$ | $\phi_H i_{1Hj}$ |
| | Natural death of Stage 1 infection | $s_{Hj} \to s_{Hj} + 1, i_{1Hj} \to i_{1Hj} - 1$ | $\mu_H i_{1Hj}$ |
| | Treatment or death from Stage 2 infection | $i_{2Hj} \to i_{2Hj} - 1, r_{Hj} \to r_{Hj} + 1$ | $\gamma_H i_{2Hj}$ |
| | Natural death of Stage 2 infection | $s_{Hj} \to s_{Hj} + 1, i_{2Hj} \to i_{2Hj} - 1$ | $\mu_H i_{2Hj}$ |
| | Infection importation | $s_{Hj} \to s_{Hj} - 1, e_{Hj} \to e_{Hj} + 1$ | $\delta s_{Hj}$ |
| Tsetse $\left(m = m_{\text{eff}}/p_H, p_H > 0\right)$ | Exposure of teneral | $s_V \to s_V - 1, e_{1V} \to e_{1V} + 1$ | $\alpha p_V (f_{H1} \left(i_{1H1} + i_{2H1}\right) / N_{H1}$<br>$+ f_{H2} \left(i_{1H2} + i_{2H2}\right) / N_{H2}) s_V$ |
| | Exposure of non-teneral, uninfected | $e_{1V} \to e_{1V} + 1, g_V \to g_V - 1$ | $\alpha p_V (f_{H1} \left(i_{1H1} + i_{2H1}\right) / N_{H1}$<br>$+ f_{H2} \left(i_{1H2} + i_{2H2}\right) / N_{H2}) \varepsilon g_V$ |
| | Progression to exposed 2 | $e_{1V} \to e_{1V} - 1, e_{2V} \to e_{2V} + 1$ | $3\sigma_V e_{1V}$ |
| | Death in exposed 1 | $s_V \to s_V + 1, e_{1V} \to e_{1V} - 1$ | $\mu_V e_{1V}$ |
| | Progression to exposed 3 | $e_{2V} \to e_{2V} - 1, e_{3V} \to e_{3V} + 1$ | $3\sigma_V e_{2V}$ |
| | Death in exposed 2 | $s_V \to s_V + 1, e_{2V} \to e_{2V} - 1$ | $\mu_V e_{2V}$ |
| | Progression to infected | $e_{3V} \to e_{3V} - 1, i_V \to i_V + 1$ | $3\sigma_V e_{3V}$ |
| | Death in exposed 3 | $s_V \to s_V + 1, e_{3V} \to e_{3V} - 1$ | $\mu_V e_{3V}$ |
| | Death in infected | $s_V \to s_V + 1, i_V \to i_V - 1$ | $\mu_V i_V$ |
| | Fed but not exposed | $s_V \to s_V - 1, g_V \to g_V + 1$ | $\alpha(1 - p_V (f_{H1} \left(i_{1H1} + i_{2H1}\right) / N_{H1}$<br>$+ f_{H2} \left(i_{1H2} + i_{2H2}\right) / N_{H2})) s_V$ |
| | Death in non-teneral uninfected | $s_V \to s_V + 1, g_V \to g_V - 1$ | $\mu_V g_V$ |

Tsetse $(m \to \infty, p_H \to 0)$

$$\frac{\mathrm{d}S_V}{\mathrm{d}t} = \mu_V N_H - \alpha S_V - \mu_V S_V$$

$$\frac{\mathrm{d}E_{1V}}{\mathrm{d}t} = \alpha p_V \left( f_{H1} \frac{I_{1H1} + I_{2H1}}{N_{H1}} + f_{H2} \frac{I_{1H2} + I_{2H2}}{N_{H2}} \right) (S_V + \varepsilon G_V) - (3\sigma_V + \mu_V) E_{1V}$$

$$\frac{\mathrm{d}E_{2V}}{\mathrm{d}t} = 3\sigma_V E_{1V} - (3\sigma_V + \mu_V) E_{2V}$$

$$\frac{\mathrm{d}E_{3V}}{\mathrm{d}t} = 3\sigma_V E_{2V} - (3\sigma_V + \mu_V) E_{3V}$$

$$\frac{\mathrm{d}I_V}{\mathrm{d}t} = 3\sigma_V E_{3V} - \mu_V I_V$$

$$\frac{\mathrm{d}G_V}{\mathrm{d}t} = \alpha \left( 1 - p_V \left( f_{H1} \frac{I_{1H1} + I_{2H1}}{N_{H1}} + f_{H2} \frac{I_{1H2} + I_{2H2}}{N_{H2}} \right) \right) S_V - \alpha p_V \left( f_{H1} \frac{I_{1H1} + I_{2H1}}{N_{H1}} + f_{H2} \frac{I_{1H2} + I_{2H2}}{N_{H2}} \right) \varepsilon G_V - \mu_V G_V$$

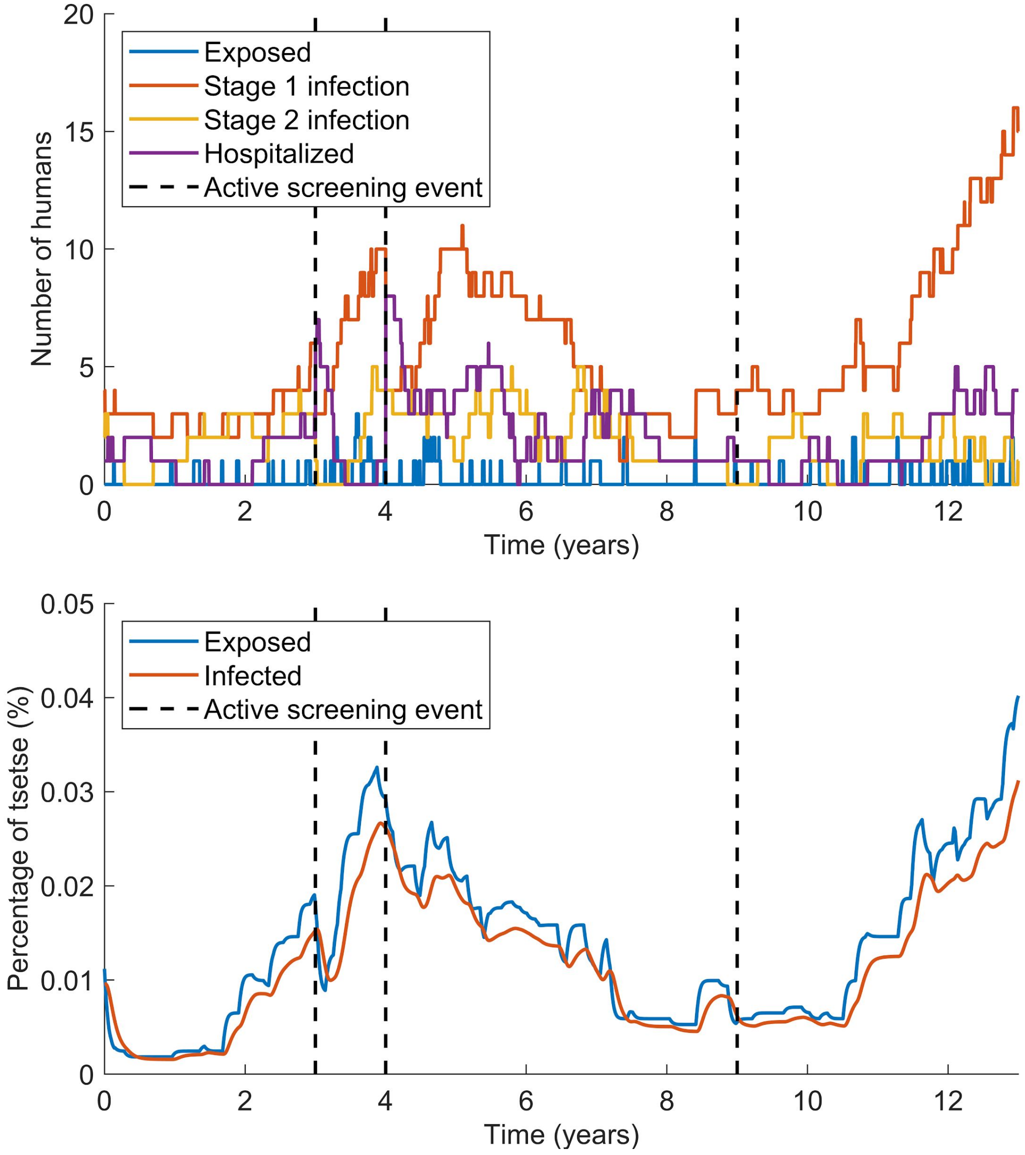


**Fig 2. A single realisation of the simulated infection dynamics of the model.** A population of 514 is used with 404, 430 and 52 of the low risk population randomly selected for active screening after three, four and nine years, respectively. Note that the number of hospitalised individuals increases at the first two active screening events, but no infected individuals are randomly selected and detected in the third screening event. We only plot those infected (or affected) by HAT, although we note that the vast majority of both populations are susceptible.

**Table 2. Model parameters.** Parameter notation and values for the stochastic model.

| Parameter | Description | Value | Source |
|---|---|---|---|
| $\mu_H$ | Natural human mortality rate | $5.4795 \times 10^{-5}$ days$^{-1}$ | [10] |
| $\omega_H$ | Human recovery rate | 0.006 days$^{-1}$ | [5] |
| $\sigma_H$ | Human incubation rate | 0.0833 days$^{-1}$ | [9] |
| $\phi_H$ | Stage 1 to 2 progression rate | 0.0019 days$^{-1}$ | [1, 2] |
| $\gamma_H$ | Exit rate from Stage 2 by treatment or death | 0.006 days$^{-1}$ | [8] |
| $N_H$ | Human population size | Varies | N/A |
| $\mu_V$ | Tsetse mortality rate | 0.03 days$^{-1}$ | [9] |
| $\sigma_V$ | Tsetse incubation rate | 0.034 days$^{-1}$ | [4, 7] |
| $\varepsilon$ | Reduced non-teneral susceptibility factor | 0.05 | [8] |
| $\alpha$ | Tsetse bite rate | 0.333 days$^{-1}$ | [11] |
| $m$ | Relative tsetse density | Varies | N/A |
| $p_H$ | Probability of human infection per single infective bite | Varies | N/A |
| $m_{\text{eff}}$ | Effective tsetse density | 6.56 | [8] |
| $p_V$ | Probability of tsetse infection per single infective bite | 0.065 | [9] |
| $f_H$ | Proportion of blood-meals on humans | 0.09 | [3] |
| $R_1$ | Proportion of low risk humans, randomly participating in screening | 0.924 | [8] |
| $R_2$ | Proportion of high risk humans, not participating in screening | 0.076 | [8] |
| $r$ | Relative bites taken on high risk humans compared to low risk | 6.6 | [8] |
| $u$ | Reporting probability for Stage 2 cases | 0.26 | [8] |
| $\delta$ | Importation of infection rate | $3.4 \times 10^{-6}$ days$^{-1}$ | fitted |

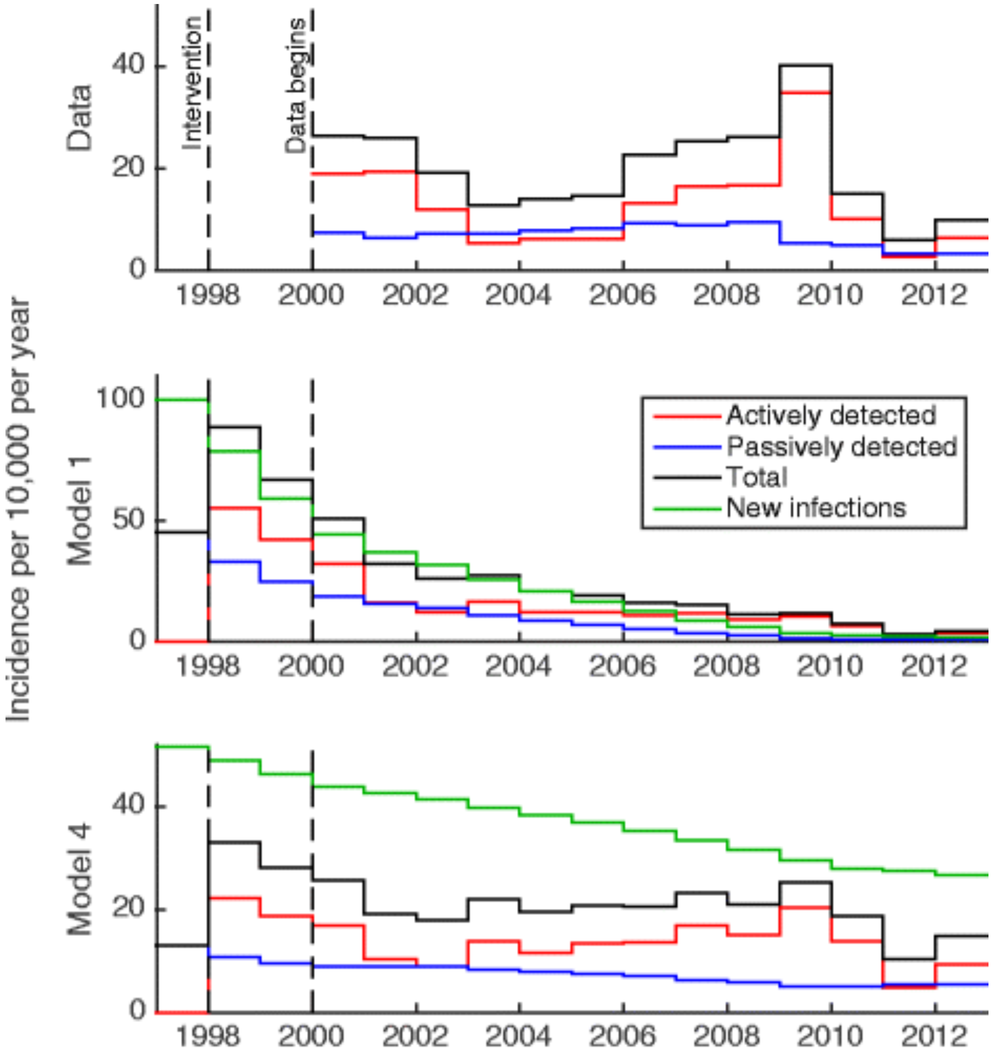


**Fig 3. Deterministic model selection.** Comparison of the reported incidence data aggregated across the study region and the corresponding model output over the years 1998–2012, for the deterministic model, both without risk structure (Model 1) and with risk structure (Model 4). Model 4 provides the better fit with the incidence data. This figure is adapted from Rock et al. [8].

## Tsetse density

One fundamental parameter of the model, the effective density of tsetse, is equal to the product of the vector-to-host ratio and the probability of human infection per single infective bite. In the original deterministic framework, these constituent terms did not need to be known, as it was only the product that was important; therefore they could not be inferred separately. However, since the stochastic model explicitly captures the number of tsetse, the two parameters are now required. Given the limited data to estimate either component parameter, we choose to explore the potential range of parameters: from very high probability of infection and hence low vector-to-host ratio, to very low probability of infection and large vector-to-host ratio such that the dynamics of tsetse can be modelled deterministically.

Considering the simplest case in the absence of disease-control measures, we find that different values for the vector-to-host ratio have a relatively limited impact on predictions of disease persistence. Unsurprisingly, large tsetse populations, which can be approximated by deterministic dynamics, lead to the greatest persistence (Fig 4); thus, throughout this paper, we utilise this worst-case scenario, although other assumptions do not change the qualitative findings.

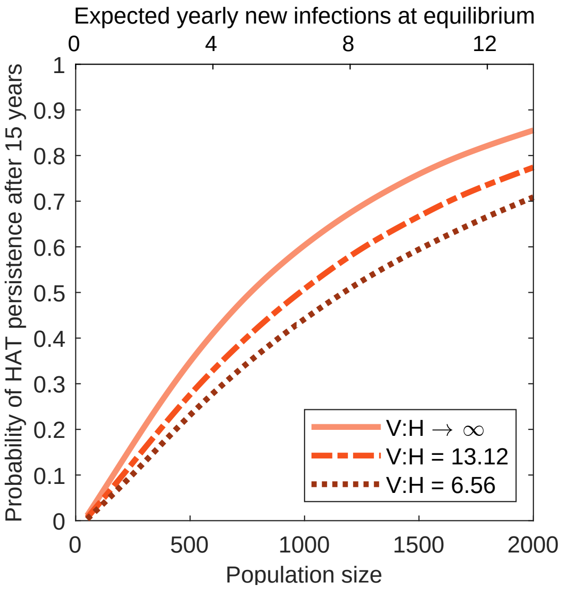


**Fig 4. Impact of the parameters forming the effective density of tsetse.** When the probability of human infection per single infective bite is high the vector-to-host ratio, V:H, is around 6.56, whereas when the probability is low, V:H becomes large and deterministic models are valid for tsetse dynamics. The expected number of yearly new infections for each population size, if the system were at equilibrium, is also given by the top scale.

## Isolated populations

For the majority of results in the main paper, we assume that importations of infection between villages do not play a significant role and so treat them independently. In many cases, such as community-level persistence, this approach is justified, as we wish to understand the stochastic dynamics in the absence of confounding imports. We also quantify the level of infectious imports by considering the probability of a village having infection present on the first screening and assuming this represents a long-term equilibrium solution, since this is the infection level before regular active screening began. By matching the WHO HAT Atlas data to the output of the model with an additional per person importation rate parameter ($\delta$) we find that, using least-squares fitting, $\delta \approx 3.4 \times 10^{-6}$ days$^{-1}$. This rate is small and so can be reasonably ignored on the studied time scales, particularly as there is some probability that this importation will not cause any subsequent transmission. In addition, it is reasonable to assume that this importation rate will decay over time, since the total case numbers in the DRC is reducing [6]. By fitting the reduction in total cases in the DRC to an exponential decay function, we get a decay rate of 0.1071, which we apply to the import rate (Fig 5).

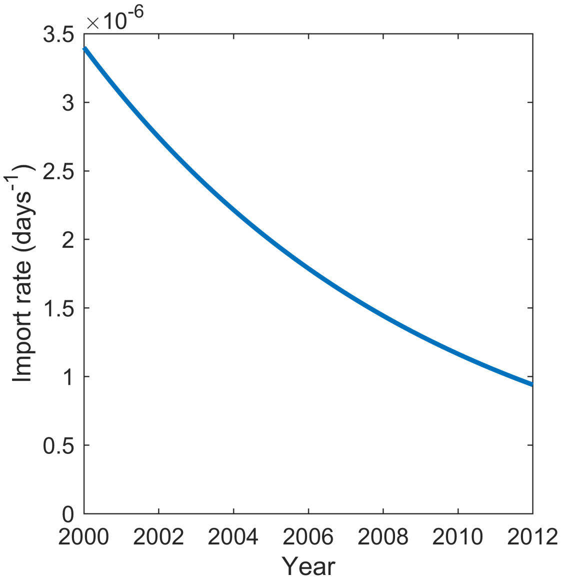


**Fig 5. Change in the importation of infection parameter in time.** The decay of the rate of importation of infection parameter is matched to the decay rate of cases of HAT in the DRC [6].

As a validation of our assumption that importations can be safely ignored over the time-scales of this study, we re-examine the match between active-screenings that fail to detect cases and model results including imports (Fig 6). This is a counter-point to Fig 2 in the main paper. We find that the inclusion of imports has an extremely small difference on the model predictions: slightly improving the fit between model and data (Fig 6A). However, under this new model, there is now an additional village where the model significantly predicts fewer zero-detections than observed, decreasing the percentage of villages within the model prediction intervals to 91.4% (Fig 6B).

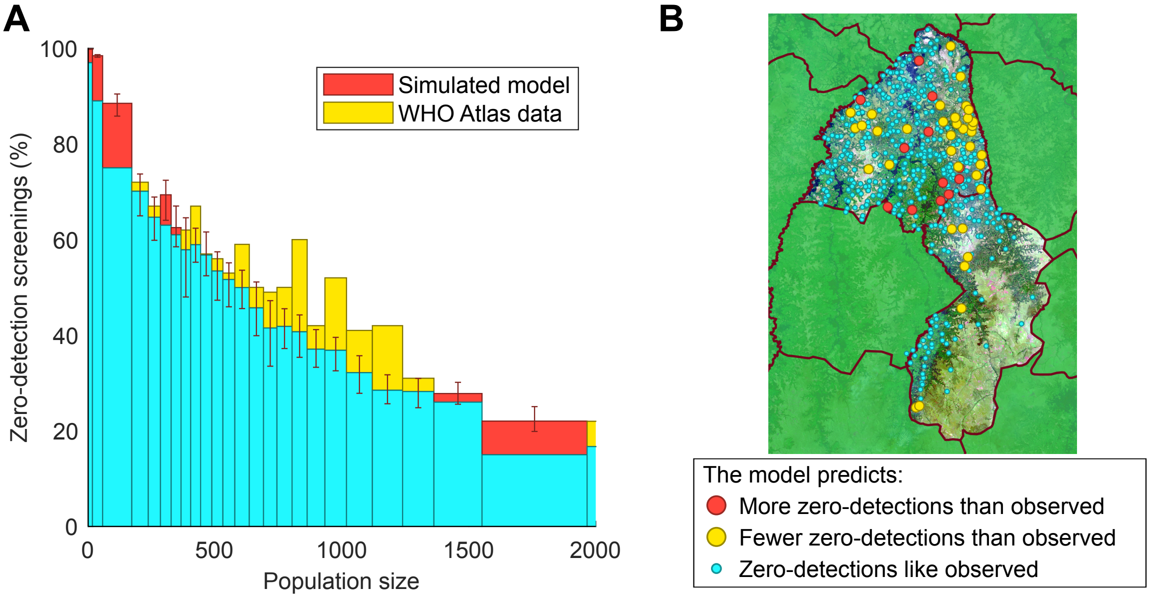


**Fig 6. Comparison of model predictions and data for active screenings with no detected cases (zero-detections) for the model with importations of infection.** (A) Histogram by population size of the percentage of active screenings that find no new HAT cases for both the model and the HAT Atlas data. The differences between this histogram and without imporatations of infection are very small. (B) Map of populations in Yasa-Bonga and Mosango showing the settlements with significant differences (at the 95% level) in the expected proportion of active screenings with no cases detected. The addition of importations of infection means this model fits the data less well, with an additional village falling outside the prediction intervals. The underlying Landsat-8 satellite map is shown courtesy of the U.S. Geological Survey.

## Population dynamics

In all model simulations, we have assumed that the population size remains constant, such that when a death occurs, the individual is replaced by a new susceptible individual immediately. We have analysed this assumption by comparing this with different population dynamics (Fig 7). We consider four additional population assumptions, all where births and deaths have been decoupled. These are: births and deaths are proportional to the number of individuals of that infection status; deaths are proportional, but births are at the constant rate from the initial equilibrium; and the same two mechanisms, but where the birth rate is increased by the population growth of 2.6%. As the populations change in size, we keep the effective tsetse density constant.

We see that the probability of gHAT persistence as time progresses is very similar for all five assumptions, while it does diverge slightly with time (Fig 7). For a population size of 2,000, after 15 years, the probability of gHAT persistence using all five assumptions gives results less than 0.01 apart and the confidence intervals all overlap. For a smaller population size, there is a bit more variation and the confidence intervals do not all overlap. The assumptions where the population size grows by 2.6% each year has a higher probability of gHAT persistence, although the maximum difference is still only approximately 0.012 and this represents a significant increase in the village population over the time span. Where there is no population growth the confidence intervals all overlap.

Thus, we can use the assumption of constant population size, since it does not significantly change the results, excluding large increases in population size for small populations, and is significantly easier to implement. Keeping the population size constant also decouples the stochastic extinction of the gHAT infection from any underlying population dynamics. If we were to introduce more elaborate

demographics into the model structure, it would be unclear if the extinction of infection was due to the stochastic process of transmission or the fluctuations in population size. Furthermore, we can associate each simulation of a village with a single population size, and can hence show probability of gHAT persistence as a function of population size.

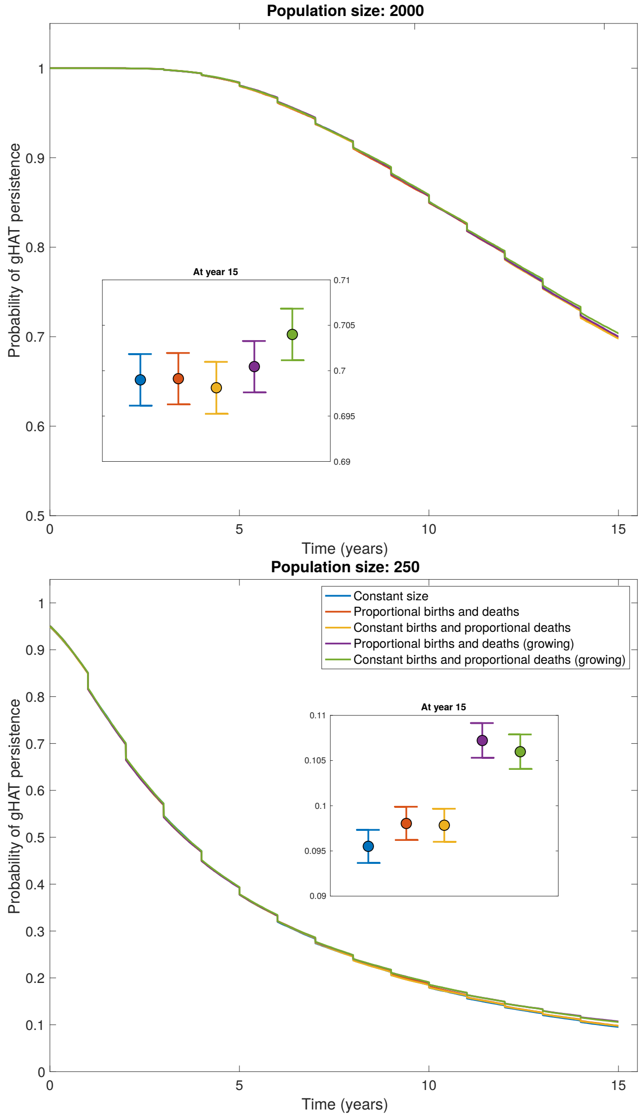


**Fig 7. Different population dynamics.** The probability of gHAT persistence is very similar for different assumptions on the population dynamics, diverging only slightly for larger times. Our typical assumption of a constant population, achieved by coupling births with deaths, provides results within the 95% confidence intervals of when births and deaths are decoupled and when births are constant. Results are shown for populations of size 2,000 and 250.

## Tau-leaping

Since we use a fixed step size for the tau-leaping algorithm of 0.1 days, Figure 8 shows that the results for persistence of HAT after 15 years are qualitatively the same for time steps of 0.01 and 1 days, so we are confident that a time step of 0.1 days is not introducing numerical errors. 95% confidence intervals are all small, in the range (0.0044, 0.0196) about the plotted mean values.

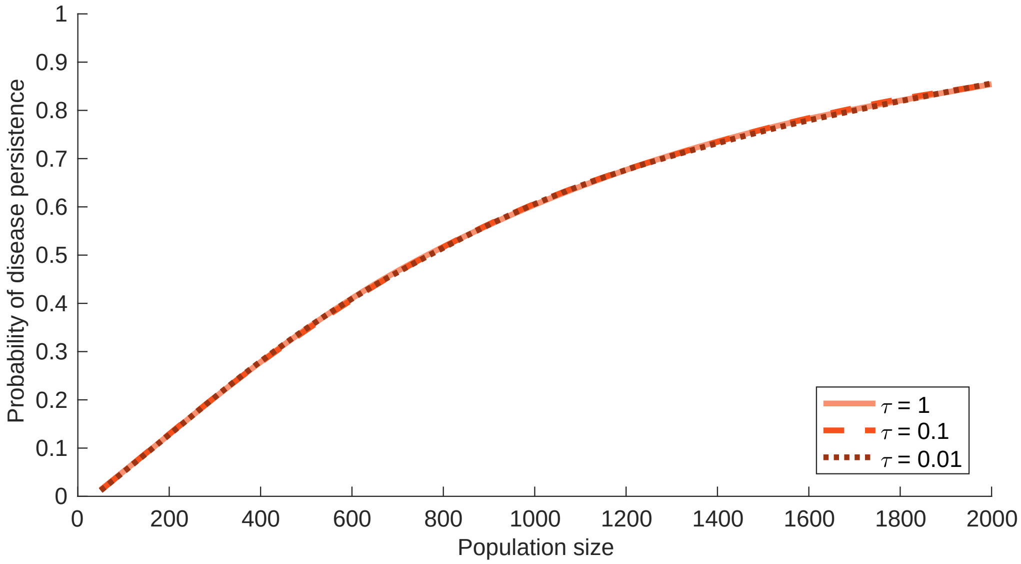


**Fig 8. Probability of disease persistence under the tau-leaping algorithm for fixed step size.** Probability of HAT persistence after 15 years for V:H $\to \infty$, simulated using three different tau-leaping time steps: $\tau = 0.01$, 0.1 and 1. Since the resulting curves are the same, the step size in the algorithm is suitable.

# Data

## Active screening data

In the main manuscript, we note that not all settlements undergo the same levels of screening. In fact, larger village populations tend to have more frequent screening, but with a lower proportion of the population tested; small populations tend to have high coverage but infrequent screens (Fig 9A and B). The smaller villages were also not typically screened in 2000–2008, while villages with populations greater than 500 were screened over the whole data period 2000–2012 (Fig 9C). We do not account for these differences in our analysis but can use these trends to help explain any discrepancies between models and the data.

In addition, the active screening data from the WHO HAT Atlas were used to calculate the mean annual screening coverage as 21%. Population sizes were taken from the census data within the WHO HAT Atlas and then modified by assuming an annual population growth of 2.6%. Coverages were then calculated as the number of people screened divided by the estimated population size, assuming a maximum coverage equal to that of the entire the low risk population size (92.4%), as described in the main manuscript. The mean annual coverage was then obtained as the mean of these coverages, where active screening occurred, as well as the 0% coverages, where no active screening was recorded. This value is thus highly dependent on the quality of the census information, as differences in population sizes will change this estimate of mean coverage. It is therefore important to have good census data for making predictions on the effect of different screening coverages.

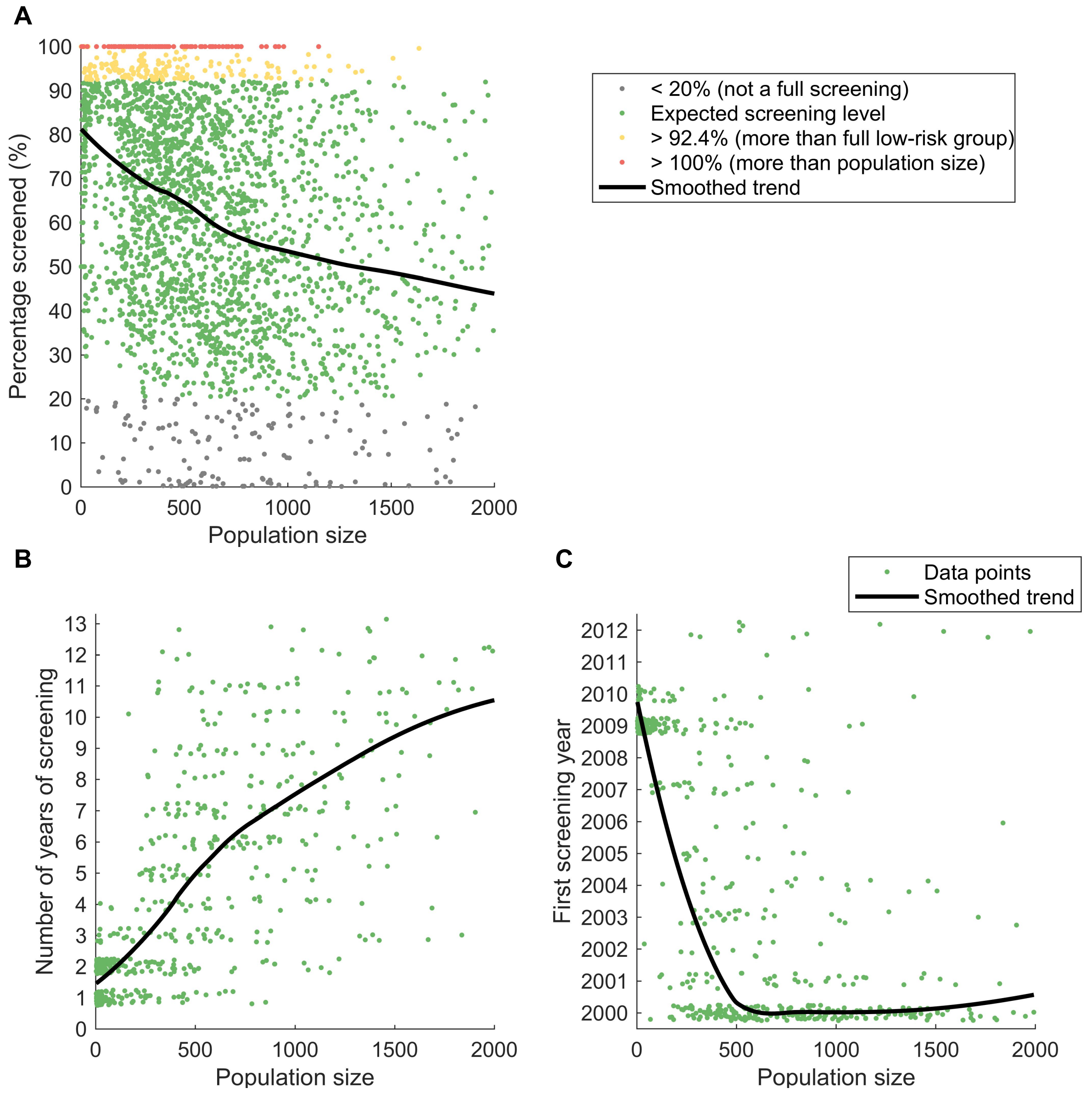


**Fig 9. Differences in screening between villages.** (A) Screening coverage in village populations. (B) Number of screenings at each settlement in the period 2000–2012. Small random perturbations are made to the integer values for visibility. (C) Years in which the first active screenings occur in the villages (within the 2000–2012 period), with small random perturbations for visibility.

# Persistence and elimination

## Local disease persistence

As a control strategy, we find that randomly selecting the screening coverage from all reported coverages slightly out-performs screening annually at the mean coverage (21%), kept fixed across all years. High levels of coverage in any one year are more likely to interrupt transmission (Fig 10A). In addition, long-term persistence requires continued transmission and so the probability of persistence decreases with time (Fig 10B and C).

## Re-invasion due to tsetse

We consider the dynamics of re-invasion, given different initial conditions to Figure 4 in the main manuscript. In a population without human infections but with endemic levels of tsetse infection, the chance that any human cases will be generated increases with settlement size (Fig 11A). This is due to tsetse being able to interact with fewer humans over their short lifespan, while new transmission events caused by human infection are less dependent on population size, as the infection will be present in the population for much longer. The probability of new transmission events is further increased by the addition of an infected human to endemic levels of tsetse infection (Fig 11B).

## Proportion of positive tests in a screening

Comparing the data on the proportion of tests that result in a positive detection of HAT to the output of the model demonstrates similar results to comparing the screenings with no detected cases, as in Figure 2 from the main manuscript (Fig 12). The difference in coverages of an active screening event mean the results have more variation than simply not detecting any new cases, yet the model is similarly capturing the behaviour seen in the data; there are a few more settlements that stand out as significantly different from the model (69 settlements, 12.3%), but these are similarly clustered where expected.

## Definition of a full screen

In calculating the probability of infection on detecting no cases, a zero-detection event is different for different screening percentages. We use 20% of the population screened to define a 'full screening'; however, similar results are achieved for the screening regime of Yasa-Bonga and Mosango 2000–2012 when 10% is used. If 50% is used, and so all screenings are of reasonable quality, there is more confidence that the same number of consecutive zero screenings are a proxy for no infection in the population (Fig 13). When screening coverage is higher and no infection is detected, there is more certainty that no infection is present.

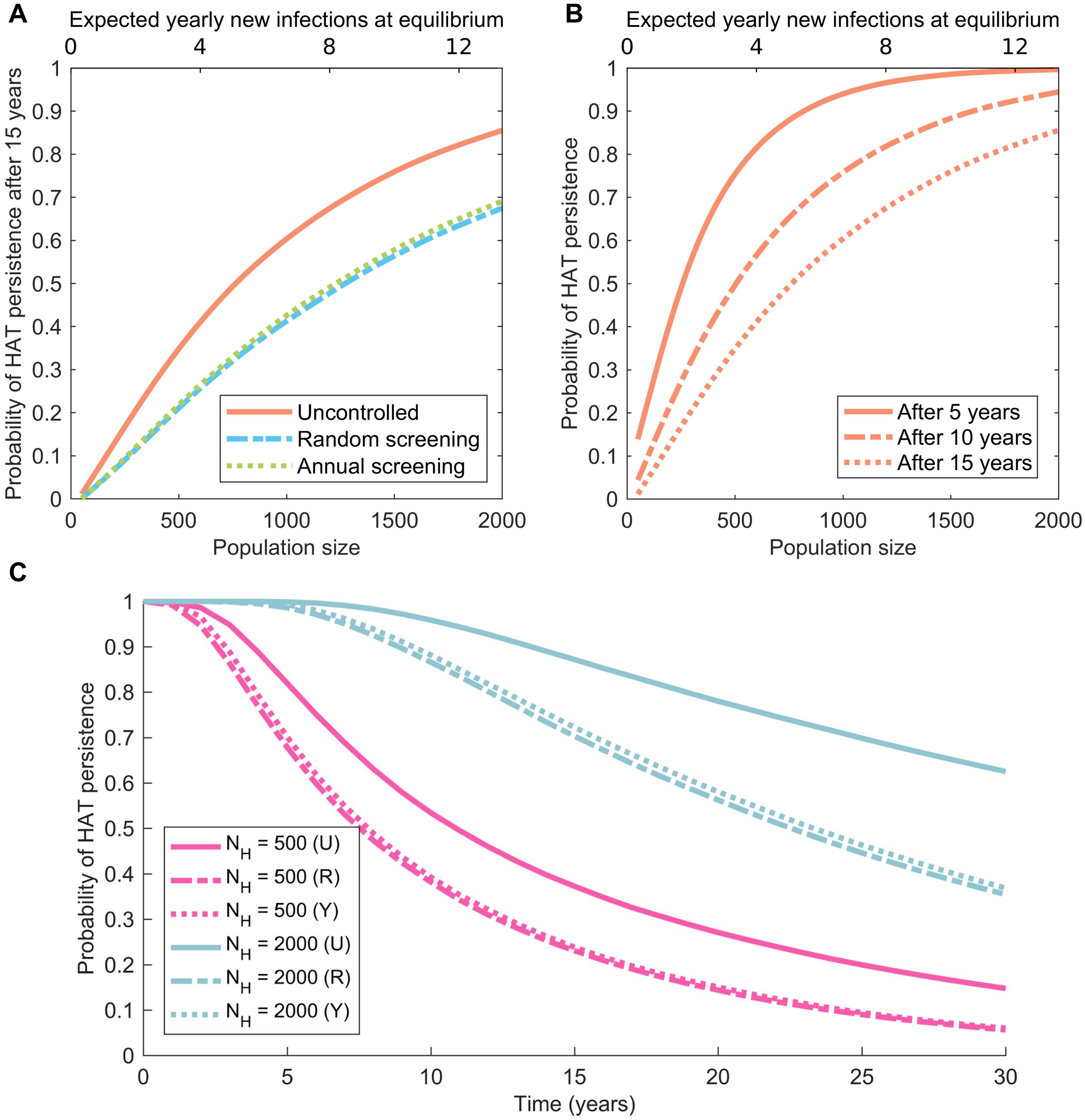


**Fig 10. Factors affecting predicted probability of HAT persistence in isolated settlements.** (A) Impact of active screening on disease persistence: both random screening (sampling from all screening coverages, including zeros) and annual screening at a fixed coverage (the mean annual coverage of 21%) yield a drop in persistence. Choosing the coverage randomly slightly out-performs screening annually at the mean coverage. (B) Probability of HAT persistence 5, 10 and 15 years after starting at the endemic equilibrium. (C) For villages with populations of sizes 500 and 2,000, the probability of HAT persistence in time for the uncontrolled setting (U), random active screening coverage (R) and annual screening at the average coverage of 21% (Y).

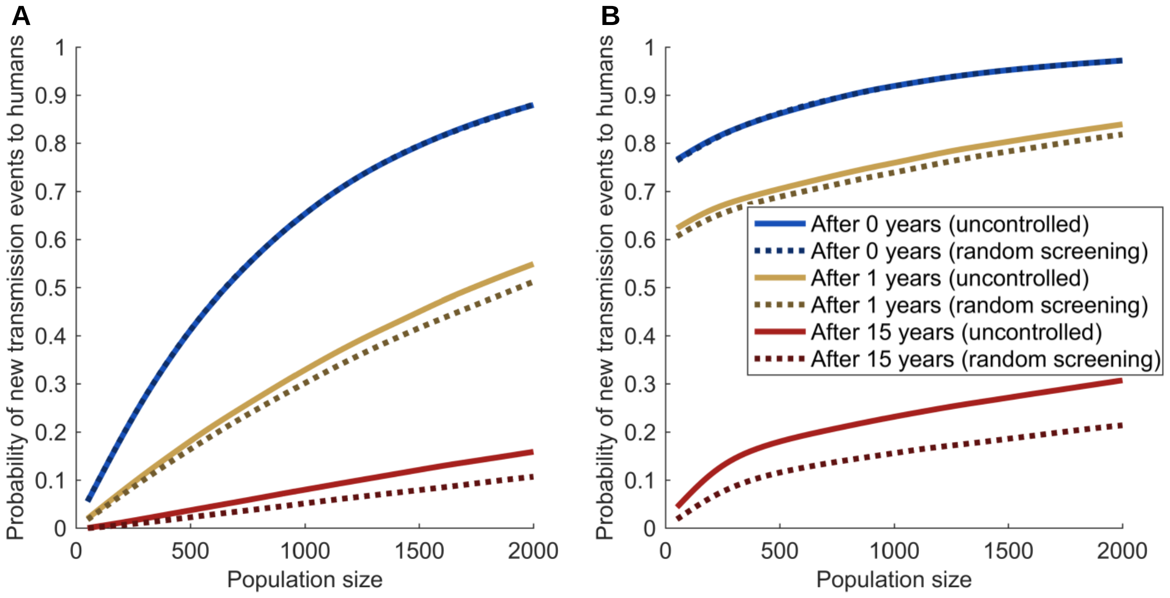


**Fig 11. Dynamics of re-invasion.** The probability that at least one human case is generated, and cases continue to be generated beyond for a given times. (A) Starting with no human infection and endemic-equilibrium levels of infected tsetse. (B) Starting with one infected human and endemic-equilibrium levels of infected tsetse.

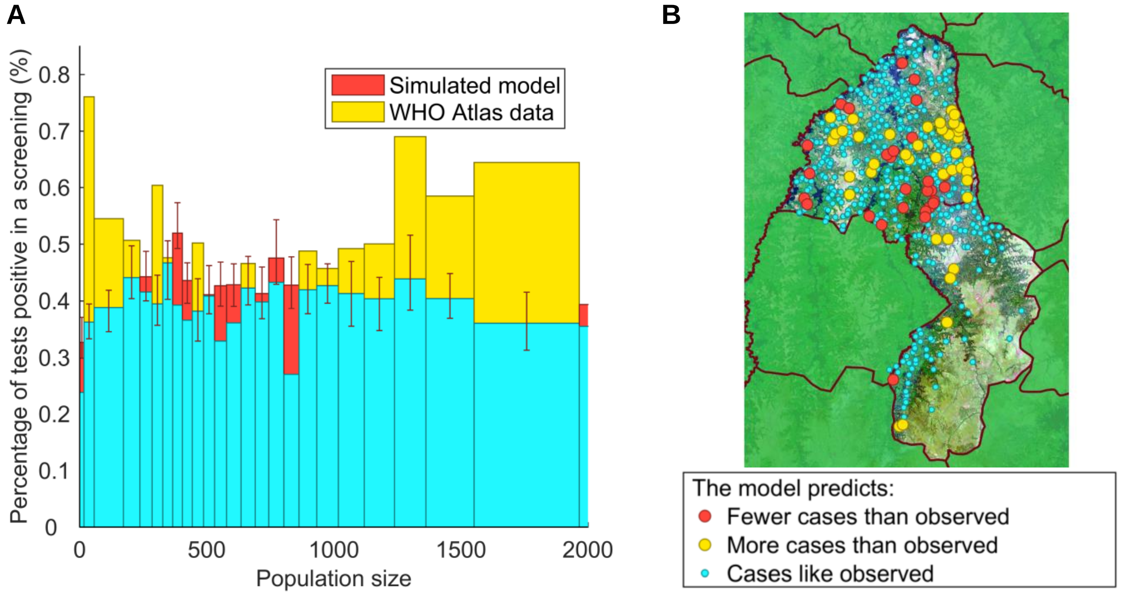


**Fig 12. Expected proportion tests that are HAT positive in an active screening.** (A) Histogram by population size of the percentage of positive tests in independent active screenings for both the model and the WHO HAT Atlas data. (B) Map of populations in Yasa-Bonga and Mosango showing the settlements with significant differences in the expected proportion of tests positive. The underlying Landsat-8 satellite map is shown courtesy of the U.S. Geological Survey.

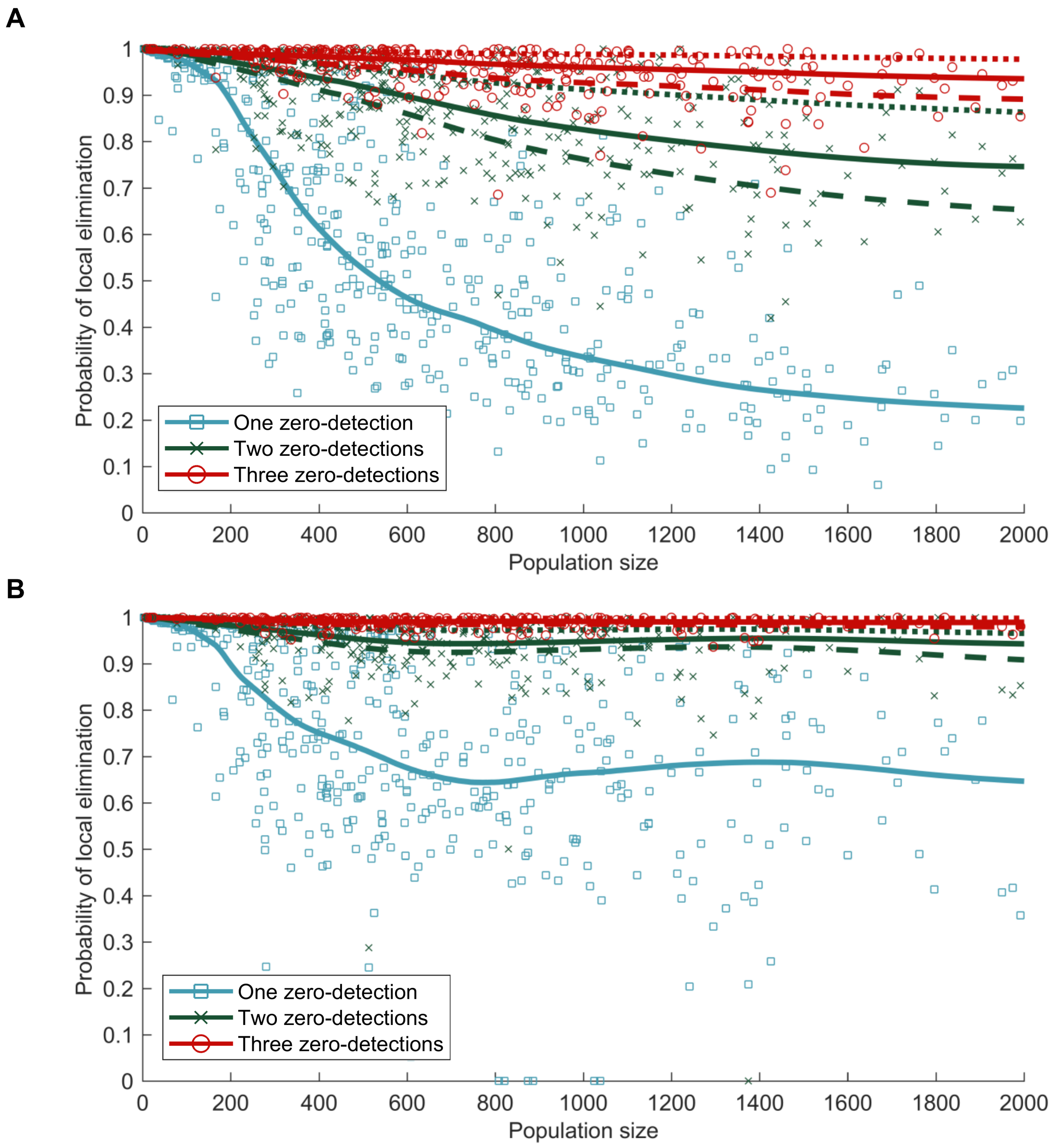


**Fig 13. Probability of elimination in a settlement given no cases have been detected in consecutive screenings.** The probability of a settlement containing no infection given that a number of consecutive active screenings detecting no cases have occurred. (A) Only active screenings where at least 10% of the population are included are used to calculate the probability. (B) At least 50% of the population must be screened for the screening to be included.

# References


[1] Francesco Checchi, João A N Filipe, Daniel T Haydon, Daniel Chandramohan, and François Chappuis. Estimates of the duration of the early and late stage of gambiense sleeping sickness. *BMC infectious diseases*, 8(1):16, 2008.

[2] Francesco Checchi, Sebastian Funk, Daniel Chandramohan, Daniel T Haydon, and François Chappuis. Updated estimate of the duration of the meningo-encephalitic stage in gambiense human African trypanosomiasis. *BMC Research Notes*, 8(1):292, 2015.

[3] PH Clausen, I Adeyemi, B Bauer, M Breloeer, F Salchow, and C Staak. Host preferences of tsetse (Diptera: Glossinidae) based on bloodmeal identifications. *Medical and Veterinary Entomology*, 12(2):168–180, 1998.

[4] S Davis, S Aksoy, and A Galvani. A global sensitivity analysis for African sleeping sickness. *Parasitology*, 138(4):516–526, 2011.

[5] Alain Mpanya, David Hendrickx, Mimy Vuna, A Kanyinda, C Lumbala, V Tshilombo, P Mitashi, O Luboya, V Kande, M Boelaert, P Lefèvre, and Pascal Lutumba. Should I get screened for sleeping sickness? A qualitative study in Kasai province. *PLoS Neglected Tropical Diseases*, 6(1):e1467, 2012.

[6] World Health Organization. Global health observatory data repository, 2018.

[7] S Ravel, P Grébaut, D Cuisance, and G Cuny. Monitoring the developmental status of Trypanosoma brucei gambiense in the tsetse fly by means of PCR analysis of anal and saliva drops. *Acta Tropica*, 88(2):161–165, 2003.

[8] Kat S Rock, Steve J Torr, Crispin Lumbala, and Matt J Keeling. Quantitative evaluation of the strategy to eliminate human African trypanosomiasis in the DRC. *Parasites & Vectors*, 8(1):532, 2015.

[9] DJ Rogers. A general model for the African trypanosomiases. *Parasitology*, 97(1):193–212, 1988.

[10] The World Bank. Data: Democratic Republic of Congo, 2015.

[11] World Health Organization. *Control and surveillance of human African trypanosomiasis*. World Health Organization, 2013.
